# Evaluation of the Flexural Strength, Water Sorption, and Solubility of a Glass Ionomer Dental Cement Modified Using Phytomedicine

**DOI:** 10.3390/ma13235352

**Published:** 2020-11-25

**Authors:** Lamia Singer, Gabriele Bierbaum, Katja Kehl, Christoph Bourauel

**Affiliations:** 1 Oral Technology, University Hospital Bonn, 53111 Bonn, Germany; bourauel@uni-bonn.de; 2 Institute of Medical Microbiology, Immunology and Parasitology, University Hospital Bonn, 53127 Bonn, Germany; g.bierbaum@uni-bonn.de (G.B.); katjakehl@o2mail.de (K.K.)

**Keywords:** conservative dentistry, glass ionomer cement, *Salvadora persica*, *Olea europaea*, *Ficus carcia*

## Abstract

Objectives: Various medicinal plant parts and extracts have been proven to be sources of biologically active compounds, many of which have been incorporated in the production of new pharmaceutical compounds. Thus, the aim of this study was to increase the antimicrobial properties of a glass ionomer cement (GIC) through its modification with a mixture of plant extracts, which were evaluated along with a 0.5% chlorohexidine-modified GIC (CHX-GIC) with regard to the water sorption, solubility, and flexural strength. Methods: *Salvadora persica, Olea europaea*, and *Ficus carcia* leaves were prepared for extraction with ethyll alcohol using a Soxhlet extractor for 12 h. The plant extract mixture (PE) was added in three different concentrations to the water used for preparation of a conventional freeze-dried GIC (groups 1:1, 2:1, and 1:2). Specimens were then mixed according to the manufacturer’s instructions and tested against the unmodified GIC (control) and a GIC modified with 0.5% chlorhexidine. Water sorption and solubility were evaluated after 7 days of immersion in distilled water. Flexural strength was evaluated in a three-point bending test after 24 h using a universal material testing machine at a crosshead speed of 1 mm/min. One-way analysis of variance (ANOVA) was used for comparison between the groups. Tukey’s post hoc test was used for pairwise comparison when the ANOVA test was significant. Results: There were no statistically significant differences between the control (M = 20.5%), CHX-GIC (M = 19.6%), 1:1 (M = 20.0%), 1:2 (M = 19.5%), and 2:1 (19.7%) groups with regard to the percentage of water sorption, while for water solubility the 2:1 (M = −0.39%) plant-modified group was significantly different from all of the other groups. Flexural strength test results showed that the 2:1 group (M = 26.1 MPa) recorded significantly higher mean values compared to all other tested groups. Conclusion and clinical relevance: The plant extracts did not negatively affect the water sorption and solubility of the GIC, while the flexural strength was improved by the addition of the plant extract at higher concentrations.

## 1. Introduction

Glass ionomer cements (GICs) belong to the class of materials known as acid-based cements. They are based mainly on three constituents, namely a water-soluble acid, ion-leachable (basic) glass, and water. GICs are commonly presented as an aqueous solution of polymeric acid and a fine glass powder, which are mixed using an appropriate method to form a viscous paste. The resulting paste is a polysalt, whereby the liquid is traditionally a mixture of acids, such as polyacrylic acid, itaconic acid, and malic acid, and the powder (glass) is the base [1]. However, alternative formulations exist, ranging from formulations were the acid is added to the glass and water is used to cause setting, to formulations in which some of the acid is blended with the glass powder and the rest is present in a dilute solution in water [2,3].

GICs are clinically attractive restorative and luting materials in different therapeutical applications in dentistry, despite the name could simply classify them as dental materials for cementation [4] Owing to their unique properties such as chemical adhesion to tooth structures and base metals, thermal compatibility with enamel, bio-compatibility and low toxicity they are largely used in dentistry, as filling materials or base materials, or alternatively to other glass materials [5,6]. However, the relatively low fracture and wear resistances are two of the major drawbacks of GICs when being compared to modern resin composite materials [7].

The durability of restorative materials is influenced by many variables, such as the hardness, water sorption, and solubility. More specifically, water sorption can change the volume of a material and can cause deterioration of the matrix structure by acting as a plasticizer [8]. The solubility of a cement has an impact on its longevity, stability, and biocompatibility. The rate of dissolution of a cement is not only affected by the testing conditions, but also by the specimen shape and thickness, powder/liquid ratio of the cement, pH, dissolution time, and concentration of the solute [9].

Moreover, flexural strength is a commonly evaluated mechanical property in GICs, which can be defined as “the ability of the material to resist deformation under load” [10]. The three-point flexural test is regarded as a simulation of a clinical situation involving the forces applied by the opposing cusp [11].

There is no perfect antibacterial filling material at the moment, and although glass-ionomers are smart materials because of their fluoride release and cariostatic properties, they are still far from optimal with regard to their antibacterial activity [12,13]. In several studies, the incorporation of antimicrobials (antibiotics, chlorhexidine, zinc) into GICs was investigated, however conflicting results were obtained relating to the influence on the physical–mechanical properties of the GICs [14,15,16].

Natural products have been used since ancient times in naturopathic medicine. Furthermore, some plant parts and extracts have been broadly used for prevention or treatment of oral diseases in dentistry [17]. Phytomedicines offer effective and promising alternatives to antibiotics for various dental diseases. They have an edge over conventional antibiotic treatments by having a great benefit-to-risk ratio [18]. Many studies have investigated these plants thoroughly, although there are approximately 500,000 plant species worldwide, of which only 1% have been phytochemically investigated [19,20].

Among the plants that show beneficial activity are *Olea europaea, Salvadora persica*, and *Ficus carcia*. *Olea europaea (O. europaea)* (the botanical name of the olive tree, which is native to Mediterranean Europe, Asia, and Africa) [21]. Different percentages of oleuropein (OL), hydroxytyrosol (HT), verbascoside, apigenin-7-glucoside, and luteolin-7-glucoside have been detected in olive leaf extracts. Both OL and HT have been shown to have anti-oxidant and antimicrobial properties [22].

*Salvadora persica (S. persica)* (also called the “toothbrush tree”) grows in the Middle East, Asia, and Africa, and is commonly used to make miswaks [23]. The tradition of using a miswak to clean the oral cavity is a part of the Greek–Arab system of medicine and is a centuries old practice. The use of miswaks for oral hygiene serves dual functions: it acts mechanically through friction between plant fibers and the tooth surface and chemically through its unique chemical composition [24].

*Ficus carcia (F. carcia)* is commonly known as the Anjir (India), fig tree (UK), or teen (Arabic). It supposedly originated from Western Asia and was spread to the Mediterranean by humans [25]. *F. carcia* leaves contain numerous active compounds, such as flavonoids, alkaloids sesquiterpenes, and saponins. These active constituents possess different antioxidant, anticancer, anti-inflammation, antiviral, and antibacterial activities [26].

When adding materials such as CHX salts or plant extracts to GICs, it is important to consider the effects that these additives may have on the mechanical and physical properties; the higher the concentration of additives, the greater the likelihood of an adverse effect [27]. Accordingly, in the present study, a conventional GIC was modified with an extract mixture of *Ficus carcia*, *Salvadora persica*, and *Olea europaea* at three different mass ratios with the aim of increasing the antimicrobial activity. The effects of the modification on the water sorption, solubility, and flexural strength was evaluated by comparing the three plant-modified groups with an unmodified negative control group and a positive control group containing 0.5% CHX.

## 2. Materials and Methods

### 2.1. Preparation of the Plant Extract Mixture

**Figure 1:**
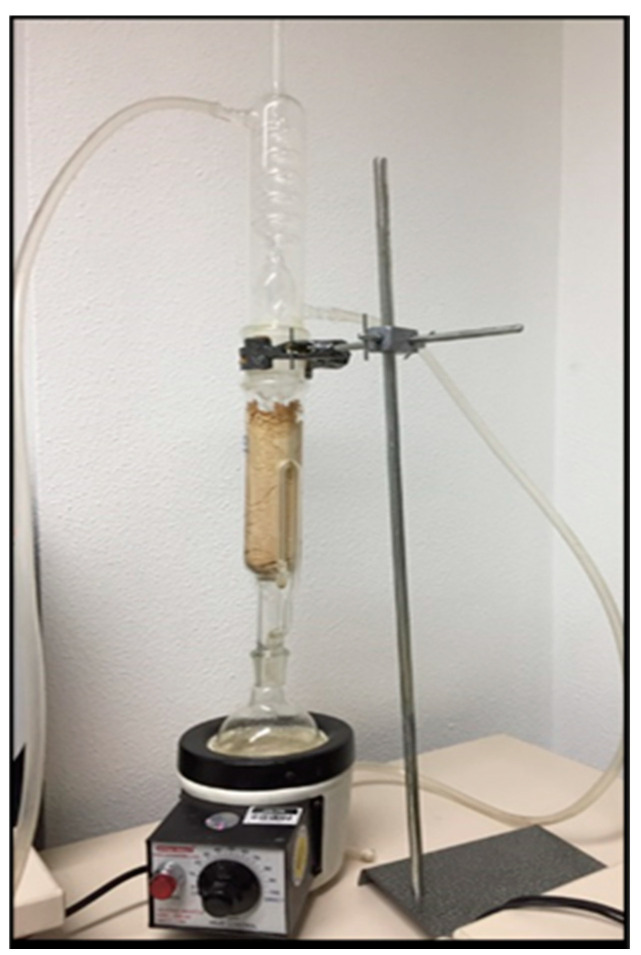
Plant extraction using alcohol in a Soxhlet extractor.

**Figure 2:**
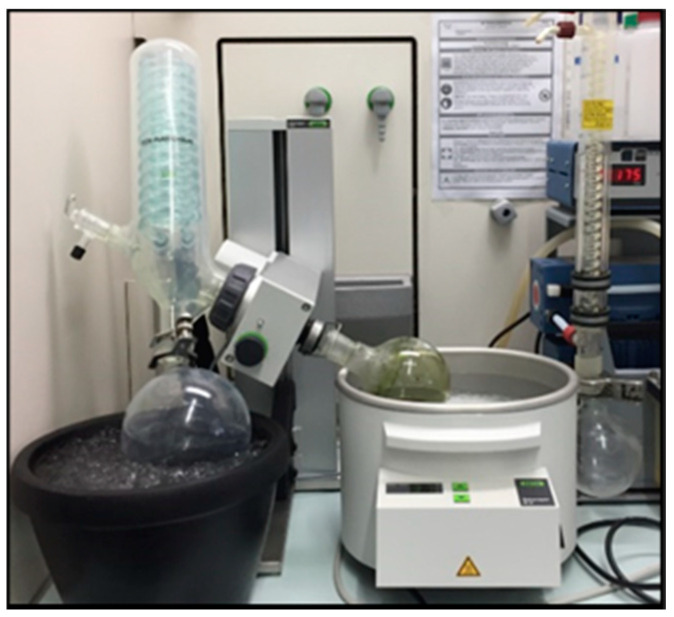
Evaporation of alcohol using a rotary evaporator.

*O. europaea, F. carcia*, and *S. persica* plants were used to prepare the extracts. Each of these plant parts was washed, dried, ground into a powder, and added to the thimble of a Soxhlet extractor (Carl Roth GmbH + Co. KG, Karlsruhe, Germany) (Figure 1). The extractions were performed using ethyl alcohol for several hours. The extraction products were then filtered and proportioned to prepare a mixture containing them all. The plant mixture was then placed at 37 °C in a rotary evaporator (Buchi Rotavapor R-300, Buchi Labor Technik GmbH, Essen, Germany; Figure 2) to remove the ethanol, leaving a crude mixture that was stored in a fridge in a closed flask at 4 °C until use [28].

### 2.2. Preparation of GIC, Extract and CHX Combinations and Specimen Grouping

The prepared plant extract mixture (PE) was added to the distilled water used for preparation of conventional GICs (Medicem aqua, Promedica GmbH, Neumuenster, Germany, Lot 1849261) at three different extract-to-water ratios (1:1, 2:1, 1:2), then mixed using a vortex mixer (CATVM4, Ingenieurbüro CAT, M. Zipperer GmbH, Ballrechte-Dottingen, Germany) to obtain a homogeneous mixture. Each concentration was stored in a sterile bottle using the exact nozzle size supplied by the manufacturer so as not to alter the original powder/liquid ratio. Each prepared liquid (plant extract + distilled water) was mixed with the glass ionomer powder component at the ratio prescribed by the manufacturer (1:2). The modified materials were grouped according to the ratio of extract added to the water, yielding three groups, then compared with a non-modified glass ionomer cement (GIC) as a negative control group and to an 0.5% CHX diacetate (*w*/*w*) (Merck KGaA, Darmstadt, Germany)-modified GIC as a positive control.

#### Specimen Grouping

The groups included the control (un-modified conventional GIC), 0.5% CHX-modified glass ionomer cement (CHX-GIC) group, and plant-modified groups (extract: water): 1:2, 1:1, 2:1.

### 2.3. Water Sorption and Solubility

In total, 50 samples of 7 mm diameter and 2 mm thickness were prepared using a Teflon mold (*n* = 10). For each group, the material was mixed according to the manufacturer’s instructions and placed inside the mold on a glass slab. A polyester matrix strip was placed over the cement surface and gently pressed with a glass slide until the mixture had set. Malformed specimens or those with voids were discarded. After one hour, specimens from each group were stored in a desiccator with silica gel (Merck KGaA, Darmstadt, Germany) for 2 h and then incubated in an oven at 37 °C for 22 h, aiming to reach constant mass, with a maximum weight difference of ±0.0005 g. Specimens were weighed on a precision analytical balance instrument (JP105DUG, Mettler-Toledo GmbH, Giessen, Germany) to obtain the initial mass (m1) values.

**Figure 3:**
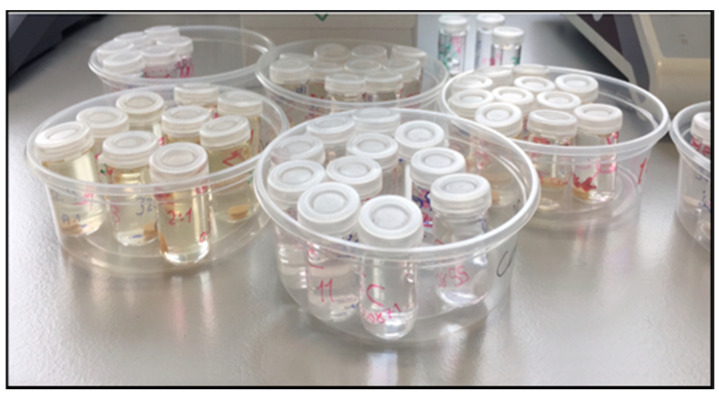
Specimens immersed in distilled water for 7 days.

**Figure 4:**
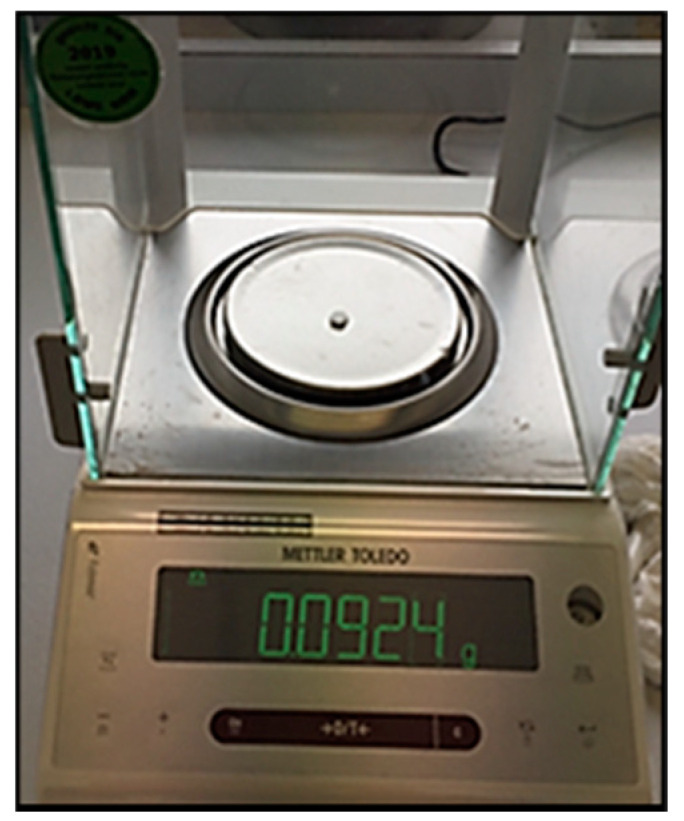
Mass determination using sensitive balance.

Each specimen was then immersed in a glass bottle containing 25 mL deionized water and was labelled for identification (Figure 3). The bottles containing the specimens were placed at 37 ± 1 °C for 7 days in an incubator. Afterwards, each specimen was taken out of the water, dried gently with a cotton pellet, and weighed again to obtain the mass values of the specimens after immersion (m2) (Figure 4). The samples were then dehydrated in an incubator at 37 ± 1 °C for 24 h and weighed for the last time to record the final mass after dehydration (m3).

The amount of water sorption was calculated from the difference between the initial mass and the wet mass (m_2_ − m_1_). The loss of material (solubility) was obtained from the difference between the initial and final drying mass values of each specimen (m_1_ − m_3_). The percentages of water sorption (Wsp) and solubility (Wsol) for each sample were calculated using the following equations [29]:

$$\begin{array}{c} W_{\mathrm{sp}}=100\cdot \frac{m_{2}-m_{1}}{m_{1}}\;,\\ W_{\mathrm{sol}}=100\cdot \frac{m_{1}-m_{3}}{m_{1}}\; \end{array}$$

### 2.4. Flexural Strength

**Figure 5:**
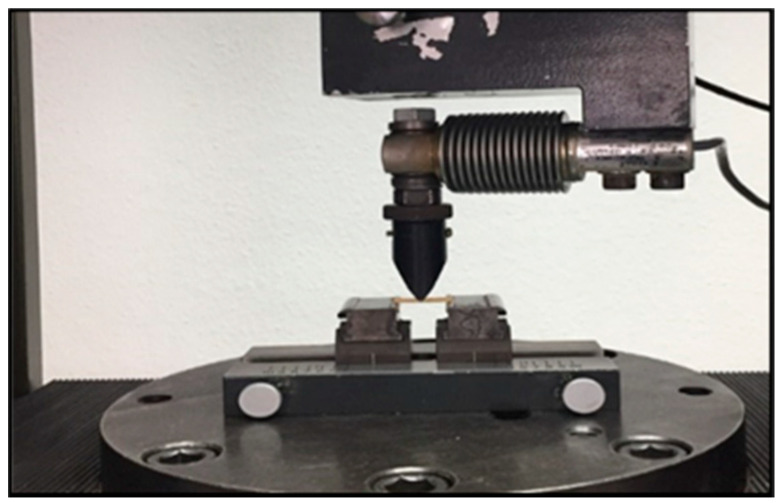
Zwick testing machine measuring flexural strength.

The flexural strength (Fs in MPa) was measured according to ISO 9917-2 using 25 × 2 × 2 mm^3^ rectangular molds (*n* = 10). Specimens were mixed according to the manufacturer’s instructions, placed inside the molds, covered with a polyethylene strip, then allowed to set. Ten minutes after setting, the specimens were removed from the molds and stored at 37 °C in a highly humid environment for 24 h. Specimens containing any voids or imperfections were discarded. The height and width of the specimens were checked using a digital micrometer to an accuracy level of 0.001 mm. The specimens were then subjected to a three-point bending test, with the distance between the two supports set at 20.0 mm [30]. The setup was integrated into a material testing machine (Zwick Zmart Pro, Zwick Roell GmbH & Co. KG, Ulm, Germany) at a crosshead speed of 1.0 mm/min (Figure 5). The **Fs** was calculated according to the following equation:

$$Fs=\frac{3\cdot F\cdot l}{2\cdot w\cdot h^{2}}\;$$

where **F** is the load at fracture, **l** is the distance between the supports (20.0 mm), **w** is the specimen width, and **h** is the specimen height [31].

### 2.5. Statistical Analysis

All variables are numerical data presented as the mean (or as the median in cases where non-parametric distribution was observed) and standard deviation (SD). The Ryan–Joiner normality test (similar to Shapiro–Wilk test) was used to examine whether or not the variables followed a normal distribution. Water sorption and solubility showed a parametric distribution. Therefore, one-way analysis of variance (ANOVA) was used for comparison between the three groups. Tukey’s post hoc test was used for pairwise comparison between the groups when an ANOVA test was significant. The significance level was set at *p* ≤ 0.05.

Observations of the flexure strength showed a non-parametric distribution, thus the Kruskal–Wallis test was used for comparison between the groups, followed by Dunn’s post hoc test for pairwise comparison. Statistical analysis was performed using Minitab 17.3.1 for Microsoft Windows (Minitab, Inc., State College, PA, USA).

## 3. Results

### 3.1. Water Sorption and Solubility

#### 3.1.1. Water Sorption

**Figure 6:**
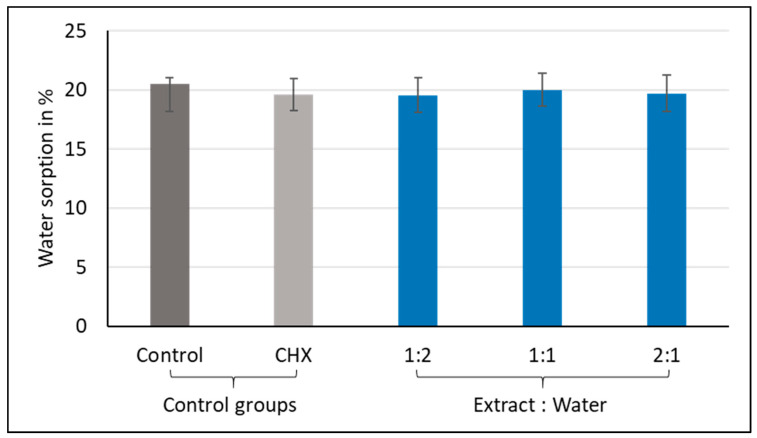
Mean water sorption values (%) showing intergroup comparison of the control, CHX, and three plant-modified groups (1:2, 1:1, 2:1).

The analysis of variance (ANOVA) indicated that there was a statistically insignificant difference in the water solubility between the groups (*p*-value = 0.908). The results are illustrated graphically in Figure 6 and the statistics are shown in Table 1. The control group had a mean value of 20.5%, while the CHX-GIC group had a mean value of 19.6%. The mean values for the plant-modified groups at ratios of 1:2, 1:1, and 2:1 were 19.5%, 20.0%, and 19.7% respectively.

#### 3.1.2. Water Solubility

**Figure 7:**
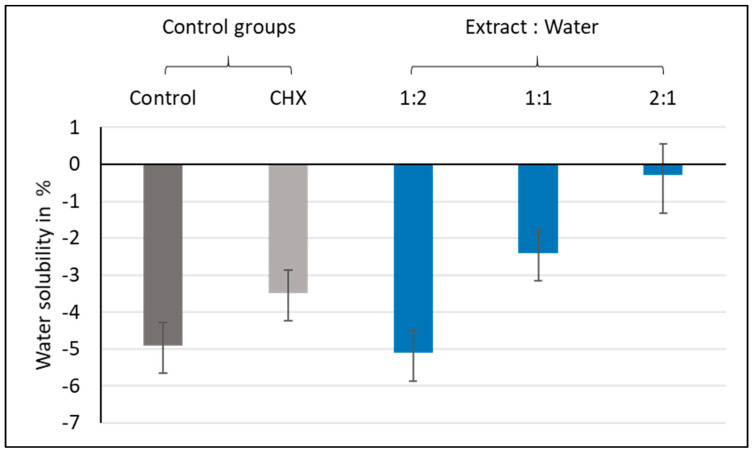
Mean water solubility values (%) showing intergroup comparisons of the control, CHX, and three plant-modified groups (1:2, 1:1, 2:1).

The analysis of variance (ANOVA) indicated that there were statistically significant differences in the water solubility between the groups, with *p*-values < 0.001. The results are illustrated graphically in Figure 7 and the statistics are shown in Table 2. The 2:1 group had the lowest negative mean value (M = −0.3%), which was significantly different from all the other tested groups, followed by the 1:1 group (M = −2.4%), then the CHX-GIC group (M = −3.5%). On the other hand, group 1:2 (M = −5.1%) and the control group (M = −4.9%) showed comparable results, with group 1:2 recording the highest negative mean value.

### 3.2. Flexural Strength

**Figure 8:**
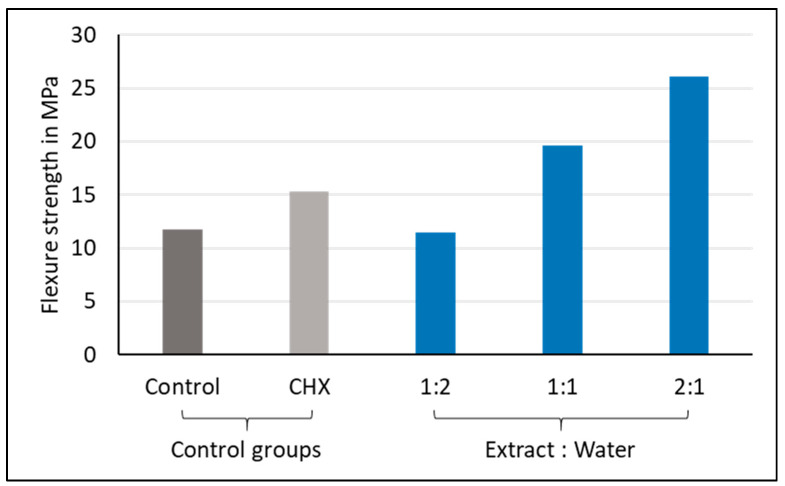
Median flexural strength values (MPa) showing intergroup comparisons of the control, CHX, and three plant-modified groups (1:2, 1:1, 2:1).

The variables showed non-parametric distribution, and thus the Kruskal–Wallis H test was used to test the effects of the plant extract mixture on the flexural strength. The results are illustrated graphically in Figure 8 and the statistics are shown in Table 3. The Kruskal–Wallis H test indicated that there was a significant effect of the plant extract mixture on the flexural strength (H (4) = 28.48, *p*-value < 0.001). Post hoc comparisons using Dunn’s test indicated that group 2:1 had the highest median flexural strength (M = 26.1 MPa), followed by group 1:1 (M = 19.6 MPa), while group 1:2 showed the lowest median flexural strength (M = 11.5 MPa). Moreover, group 2:1 was statistically different from the control (M = 11.8 MPa), CHX-GIC (M = 15.3 MPa), and 1:2 (M = 11.5 MPa) groups.

## 4. Discussion

The great potential for exploring natural anti-microbial compounds came from the increasing resistance of many pathogens to currently used agents, such as antibiotics and antiviral agents [32]. Although many natural antibacterial agents have shown effective results against cariogenic salivary flora when used in mouthwashes or toothpastes, there is still a lack of data regarding the effects of their addition on the properties of glass ionomer cements [33,34].

In the present study, an alcoholic extract mixture of *Salvadora persica*, *Ficus carcia*, and *Olea europaea* was prepared using a Soxhlet extractor. The extract mixture was then used to modify a conventional freeze-dried GIC by adding it to the water used for cement preparation at three different extract-to-water mass ratios (1:1, 1:2, 2:1). The extract-modified materials were evaluated and compared with an unmodified GIC (Control) and 0.5% CHX-modified GIC (CHX-GIC) with regard to the water sorption, solubility, and flexural strength.

Although CHX is considered the gold standard for antibacterial applications, its incorporation in GICs frequently results in changes in the physical and mechanical properties [14,35]. This is probably the reason why the combination of chlorhexidine and other antibacterial substances with GICs has still not been incorporated into their production. Here, 0.5% of CHX (*w*/*w*) was chosen in the current study to be added to the GIC powder, based on reports that stated that this percentage might be the best option, since the antibacterial activity increased and the physical–mechanical properties were not compromised at this percentage [36,37,38].

### 4.1. Water Sorption and Solubility

Water sorption and solubility are critical parameters in the evaluation of bonding materials and are directly related to the longevity of a cement. Water sorption tests actually measure the net gain in weight of a specimen resulting from diffusion of water molecules and elution of monomers and other small molecules [39].

Two well-known theories could explain the diffusion of water through polymeric materials: one is the free volumetric theory, whereby the water diffuses through microvoids without any mutual relationship with the polar molecules in the material. The other theory is called the interaction theory, whereby water diffuses through material to bind successively to the hydrophilic groups. If there is a negative correlation between the diffusion and equilibrium water uptake, the latter pattern of diffusion supposedly occurs [39].

Initially within GICs, the water sorption process transports calcium and aluminum ions to react with polyacrylic acid. However, over time, excessive water uptake can cause deterioration and degradation of the cement, resulting in impaired structural and mechanical properties [40]. In the present study, the water sorption and solubility were measured after immersion of the specimens for 7 days, because in several previous studies it was stated that the maximum amount of water gain occurs within the first week in most hydrophilic materials [41,42].

For water sorption, all of the tested groups showed water gain at the end of the immersion period. The hydrophilicity of a polymer is determined by its chemistry, polymerization linkages, and the presence of hydroxyl, carboxyl, or phosphate groups, which make them more hydrophilic and more prone to water sorption. Moreover, crack lines were also observed in many specimens of the different groups, which might be partly responsible for the high water sorption values. Kucukyilmaz et al. in 2016 investigated the microleakage scores of GICs and concluded that the observed crack areas and lines in many of the samples resulted in higher rates of dye penetration, which might be the reason for the variations in values from earlier studies [43].

Moreover, the results showed no statistically significant differences between mean values for the control (M = 20.5%), CHX-GIC (M = 19.6%), and the three extract modified groups (1:1, M = 20.0%; 1:2, M = 19.5%; 2:1, M = 19.7%) (see Figure 6). A possible explanation is that there is no variation in their chemical composition, as the tested groups are all basically conventional GICs, and consequently there were no differences in their water sorption capacity values either [44].

Solubility is the ability of a substance to dissolve in another substance, expressed as the concentration of saturated solution of a solvent in a dissolvent [45]. For water solubility, all of the tested groups showed negative mean values (control, M = −4.9%; CHX-GIC, M = −3.5%; 1:1, M = −2.4%; 1:2, M = −5.1%; 2:1, M = −0.3%) (see Figure 7). Negative solubility values may be attributed to incomplete dehydration of these materials, which does not mean that no solubility occurred in these materials, but may hint to their solubility. Negative values were also reported by Toledano et al. in 2006, Keyf et al. in 2007, and Sinthawornkul at el. in 2017 [46,47,48]. An explanation could be that the acid–base reaction was prolonged and water molecules were continuously bonded into their structures. Therefore, the materials gained weight and expanded [48]. On progression of the acid–base reaction, the GIC takes up water as an integral part of its structure; therefore, the longer the rate of the reaction, the greater the water uptake into the structure of the cement, and vice versa [46].

In addition, there were statistically significant differences among the tested groups—the control and 1:2 groups showed the highest negative mean values, followed by the CHX-GIC and 1:1 groups, while the 2:1 group showed the lowest negative mean values. Such differences could mean that the water molecules did not bond to the structure equally in all groups after the acid–base reaction ended. Therefore, some of the absorbed water molecules were either only trapped in the space of the matrix, filler, or matrix–filler interface. Then, this loosely bonded water was vaporized out of the sample after drying in the desiccator [40].

### 4.2. Flexural Strength

Flexural strength was chosen for evaluation because it is more sensitive to small changes in a material’s structure than the compressive strength and allows the clinical loading situation to be mimicked by giving an appropriate estimate of the tensile strength of a material [49]. However, it is difficult to prepare the beam specimens required for the test without flaws or cracks [50].

Flexural strength was measured according to ISO 9917-2. The test was performed after 24 h of storage, as a GIC’s final setting and strength are achieved after 24 h, and they usually present lower strength values during the first hours [50,51]. The values demonstrated in the present study were comparable to the results presented by Kutuz et al. in 2019 [52] and Sajjad et al. in 2019 [53]. The results showed that the plant extract enhanced the flexural strength of the GIC, with the 2:1 group (M = 26.1 MPa) having the highest median flexural strength, which was statistically different from the control (M = 11.8 MPa), CHX-GIC (15.3 MPa), and 1:2 groups (11.5 MPa) (see Figure 8). Moreover, his effect was found to be concentration-dependent, whereby the 2:1 group yielded the highest flexural strength value, followed by the 1:1 group (M = 19.6 MPa); both were significantly different from the control, CHX-GIC, and 1:2 (lowest extract concentration) groups.

This was explained through the chemical analysis of the plant extract, which revealed the presence of cinnamic and bornyl acetic carboxylic acids. It is expected that by adding these acids to glass ionomer liquids, the degree of cross-linking increases, together with polysalt bridge formation, which subsequently strengthen the mechanical properties of the cement. This is in accordance with Prentice et al., who showed in 2006 that lowering the pH by increasing the concentration of polyacrylic acid with another carboxylic acid improves the release of ions from the surface of the glass ionomer powder and increases the rate of cross-linking [54]. Another explanation that was suggested by Yup et al. in 2001, Behr et al. in 2006, and Moshaverinia et al. in 2008 is that the extract could affect the amount of unreacted powder particles within the matrix, which may act as reinforcing fillers, impeding crack propagation within the cement [55,56,57].

The test and investigation were done in vitro, but the results greatly contribute to the understanding of the concept of GICs as smart materials rather than as polymeric resin composite materials in dentistry. It remains to be seen if the addition of alcoholic molecules from plants can improve or decrease the wettability of dental tissues in vivo. The adhesion of these materials clinically depends on the linking of COO- -to CA++ in the tooth. However, the drying conditions following alcohol application can critically modify this adhesion and can lead to failure of the restoration, which depends not only on the antibacterial properties of the material itself (GIC), but also on the strength of the bond (gap-free) achieved between the dentine or enamel and the GIC [58].

The limitation of this study is that the testing conditions did not simulate the exact clinical situations. Factors such as the clinical conditions, degree of moisture contamination, powder–liquid ratio, mixing technique, manufacturer, and the batch of the luting cement usually affect the physical and mechanical behaviours of dental cements [59]. In addition, in order to evaluate the flexural strength, only static forces are considered, but not the complex dynamic forces that result in restoration in the oral cavity. However, our in vitro results are important for screening of the sorption, solubility, and flexural strength after the modification of GICs with plant extracts. Other variables could alter the results of the present report. In fact, acidic environments [60] and wear [61] can alter the surface characteristics of dental materials. Therefore, in the future, further studies are needed on the topic.

## 5. Conclusions

Within the context of this study, it can be concluded that the addition of a plant extract mixture in an attempt to improve the antimicrobial properties of the GIC enhanced the flexural strength, without compromising water sorption and solubility behavior of the GIC.

## Figures and Tables

**Table 1:** Results of ANOVA test for water sorption of control, CHX, and three plant-modified groups (1:2, 1:1, 2:1).

Group	N	Mean %	St Dev	*p*-Value *
Control	10	20.5	2.7	0.908
CHX-GIC	10	19.6	1.2	
1:2	10	19.5	2.6	
1:1	10	20.0	1.3	
2:1	10	19.7	4.3	

* Significant at *p* ≤ 0.05.

**Table 2:** Results for the ANOVA and Tukey post hoc test for water solubility values for the control, CHX, and three plant-modified groups (1:2, 1:1, 2:1).

Group	N	Mean %	St Dev	*p*-Value *		Pairwise Comparison **	
Control	10	−4.9	1.5	<0.001	A		
CHX-GIC	10	−3.5	1.0			B	
1:2	10	−5.1	0.6		A		
1:1	10	−2.4	0.9			BC	
2:1	10	−0.3	1.4				D

* Significant at *p* ≤ 0.05. ** Groups that do not share a letter are significantly different.

**Table 3:** Results of Kruskal–Wallis H test and pairwise comparison for the flexural strength values of the control, CHX, and three plant-modified groups (1:2, 1:1, 2:1).

Variable	N	Median (MPa)	IQR (MPa)	*p*-Value *	Pairwise Comparison **		
Control	10	11.8	5.1	<0.001	A		
CHX-GIC	10	15.3	10.3		A	B	
1:2	10	11.5	5.9		A	B	
1:1	10	19.6	4.6			B	C
2:1	10	26.1	9.0				C

* Significant at *p* ≤ 0.05. ** Medians that do not share a letter are significantly different.
